# Association between caffeine and the eGFR dip after initiation of SGLT2 inhibitors in adult patients

**DOI:** 10.14814/phy2.70638

**Published:** 2025-10-27

**Authors:** Zach Kisley, Amanda J. Kalishman, Aliza A. Memon, Sandeep S. Dhindsa, John C. Edwards, Amy Mosman, Krista L. Lentine, Paul Kunnath, Emily Wood, Kana N. Miyata

**Affiliations:** ^1^ Department of Internal Medicine Saint Louis University St Louis Missouri USA

**Keywords:** adenosine, caffeine, estimated glomerular filtration rate, sodium‐glucose cotransporter‐2 inhibitors, tubuloglomerular feedback

## Abstract

Sodium‐glucose cotransporter‐2 inhibitors (SGLT2i) are cornerstone therapies for heart failure and chronic kidney disease. Clinical trials have demonstrated that the estimated glomerular filtration rate (eGFR) typically declines within the first few weeks after SGLT2i initiation. One proposed mechanism of the acute eGFR dip is the reduced intraglomerular pressure through tubuloglomerular feedback (TGF). Adenosine is a key mediator in TGF signaling, and caffeine, a nonselective adenosine receptor antagonist, may influence this process. We conducted a retrospective cohort study at SSM Health Saint Louis University Hospital to examine whether caffeine intake influences the initial eGFR dip following SGLT2i initiation. Eligible patients completed a caffeine consumption survey, and chart reviews assessed creatinine and proteinuria at baseline and 1 month post‐initiation. Data from 62 patients (mean age 60.1 ± 12.4 years; median eGFR 50.0 [IQR 37.2–66.9] mL/min/1.73 m^2^) revealed a negative correlation between caffeine intake and eGFR dip at 1 month (*r* = −0.31, *p* = 0.02). Multivariable regression showed caffeine intake and baseline creatinine were independently associated with the eGFR dip. These findings suggest high caffeine consumption may attenuate the early eGFR decline seen with SGLT2i therapy. Further research is warranted to explore its impact on long‐term renal outcomes.

## Graphical abstract

A retrospective study showed that high caffeine intake was associated with an attenuated initial eGFR dip following the initiation of an SGLT2 inhibitor.

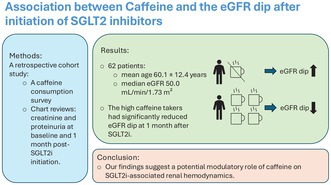

## 1 INTRODUCTION

Sodium‐glucose cotransporter‐2 inhibitors (SGLT2i), initially approved as anti‐hyperglycemic agents, have recently emerged as cornerstone therapies for heart failure and chronic kidney disease (CKD). This shift in clinical application is supported by a growing body of evidence indicating that SGLT2i not only improve glycemic control but also confer significant benefits in terms of kidney function, cardiovascular outcomes, and mortality reduction (Heerspink et al., 2020; Herrington et al., 2023; McMurray et al., 2019; Packer et al., 2020; Zinman et al., 2015).

Multiple clinical trials have shown that the estimated glomerular filtration rate (eGFR) initially declines within several weeks after initiation of SGLT2i accompanied by a decrease in proteinuria, followed by a slower decline of eGFR compared to placebo in the long term (Heerspink et al., 2020; Herrington et al., 2023; Zinman et al., 2015). One proposed mechanism of the acute eGFR dip is the reduced intraglomerular pressure caused by afferent arteriolar vasoconstriction and efferent arteriolar vasodilation through tubuloglomerular feedback (TGF) (Heerspink et al., 2016; Thomson & Vallon, 2021). SGLT2 inhibition, by blocking a fraction of the sodium reabsorption at the proximal tubule, increases the sodium chloride delivery to the macula densa, leading to more adenosine triphosphate (ATP) release and breakdown to adenosine (Cherney et al., 2014; Thomson et al., 2012; Vallon et al., 1999). Basolateral release of adenosine binds to adenosine A1 receptors (A1AR) on vascular smooth muscle cells lining afferent glomerular arterioles, promoting the afferent arteriole constriction (Cherney et al., 2014). In addition, it induces efferent arteriolar vasodilation via adenosine A2 receptors (Ren et al., 2001). Accordingly, increased adenosine release can decrease intraglomerular capillary hydrostatic pressure and lead to a reduced glomerular filtration rate (GFR) (Schnermann, 2002).

Caffeine is a non‐selective adenosine receptor antagonist (Daly et al., 1994). Caffeine consumption has a well‐known diuretic effect, potentially caused at least in part by blocking TGF and increasing the GFR. A previous animal study showed that caffeine elicited a diuresis and natriuresis in wild‐type (WT) mice but not in A1R knockout (KO) mice, suggesting its adenosine‐mediated effect (Rieg et al., 2005). In this study, we examined the hypothesis that individuals with high caffeine consumption exhibit a blunted eGFR dip following the initiation of SGLT2i compared to those with little or no caffeine intake.

## 2 MATERIALS AND METHODS

### 2.1 Study design

This is a single center cohort study performed at SSM Saint Louis University from February 2022 to February 2024. After written informed consent was obtained, participants completed a survey regarding their daily caffeine consumption at the time of SGLT2i initiation. Retrospective chart review was performed to collect demographics and laboratory data, including serum creatinine levels and proteinuria or albuminuria before and 1 month after SGLT2i initiation. This study was reviewed and approved by the Saint Louis University Institutional Review Board and was conducted in accordance with the Declaration of Helsinki.

### 2.2 Study population

Patients visiting Internal Medicine, Endocrinology, and Nephrology clinics were pre‐screened to determine their eligibility. Eligible patients were 18 years or older, those on an SGLT2 inhibitor (dapagliflozin, empagliflozin, or ertugliflozin) for at least 2 months, those who had creatinine measurements within 6 months before as well as 1–2 months after the SGLT2i initiation. Exclusion criteria included those with eGFR <20 mL/min/1.73m^2^ at the time of SGLT2i initiation or those with renin‐angiotensin‐aldosterone system (RAAS) blockade initiated at the same time as the SGLT2i. Eligible participants were given a list of caffeinated substances and asked to fill out the number of times and amount they consumed of those in a day. Survey answers were put into a caffeine calculator which was based on standards and averages of caffeine content from three different independent sources (Caffeine Informer, 2025; Centre For Science in the Public Interest, 2025; Mayo Clinic Healthy Lifestyle, 2025).

### 2.3 Chart review

Demographic and renal function data were extracted from the patient's medical records. Information collected included age, sex, race/ethnicity, comorbid conditions, medications at the time of SGLT2i initiation, and laboratory values. Laboratories included the baseline creatinine level measured within 6 months before SGLT2i initiation, post‐SGLT2i creatinine measured within 1–2 months after SGLT2i initiation, UPCR (urine protein‐to‐creatinine ratio) or UACR (urine albumin‐to‐creatinine ratio) before SGLT2i initiation and 1–2 months after SGLT2i initiation.

### 2.4 Outcome measures

eGFR was calculated using the 2021 race‐free CKD‐EPI_creatinine_ equation (Inker, 2021). eGFR dip (%) at 1 month was calculated by (pre‐SGLT2i eGFR − post‐SGLT2i eGFR) × 100/(pre‐SGLT2i eGFR). Proteinuria or albuminuria reduction were calculated by (pre‐SGLT2i UPCR − post‐SGLT2i UPCR) × 100/(pre‐SGLT2i UPCR) or (pre‐SGLT2i UACR − post‐SGLT2i UACR) × 100/(pre‐SGLT2i UACR), respectively.

### 2.5 Statistical analysis

Statistical analyses were performed using the statistical software package SAS Studio (SAS Institute, Cary, NC). Spearman correlation was used for nonparametric data analysis. eGFR dip at 1 month between the three groups based on caffeine consumption was analyzed by the Kruskal–Wallis test for skewed distribution. We used univariable linear regression analysis to assess the variables associated with eGFR dip. We further performed multivariable linear regression analysis to identify variables independently associated with eGFR dip. The association of initial eGFR dip with clinical parameters were explored using multivariable linear regression analysis, adjusting for variables that were identified in univariable analysis to be significantly correlated with eGFR dip. *p* < 0.05 was considered statistically significant.

## 3 RESULTS

In total, 62 eligible patients agreed to participate in the caffeine intake survey. Patient characteristics are listed in Table 1. Participants' mean age was 60.1 ± 12.4 years and median eGFR was 50.0 (IQR 37.2–66.9) mL/min/1.73 m^2^. Among the cohort, 77.4% had diabetic kidney disease. As expected, eGFR for the entire population decreased by 10.8 ± 13.1% at 1 month after the initiation of SGLT2i.

**TABLE 1:** General characteristics of the participants.

Baseline characteristics	*N* = 62
Age, years, mean (SD), range	60.10 (12.41), 25–83
Gender: male, *n* (%)	32 (51.6)
Race, *n* (%)	
Black	36 (58.1)
Caucasian	26 (41.9)
CKD etiology, *n* (%)	
Diabetes mellitus	48 (77.4)
Hypertension	8 (12.9)
Glomerulonephritis	6 (9.7)
Blood pressure, mmHg, mean (SD)	
Systolic	134.10 (16.00)
Diastolic	78.18 (10.47)
SGLT2 inhibitor, *n* (%)	
Empagliflozin	45 (72.6)
Dapagliflozin	16 (25.8)
Ertugliflozin	1 (1.6)
Caffeine intake, mg/day, median [IQR]	75.50 [3.75–271.50]
Serum creatinine level, mg/dL, median [IQR]	1.4 [1.1–1.7]
eGFR, mL/min/1.73 m^2^, median [IQR]	50.0 [37.2–66.9]
Urinary protein‐to‐creatinine ratio, g/g, median [IQR], *n* = 17	0.66 [0.13–3.32]
Urinary albumin‐to‐creatinine ratio, mg/g, median [IQR], *n* = 29	33 [7–614]
Medication use, *n* (%)	
ACEi/ARBs	44 (71.0)
Diuretics	33 (53.2)

Abbreviations: ACEi, angiotensin‐converting enzyme inhibitor; ARB, angiotensin receptor blockers; CKD, chronic kidney disease; eGFR, estimated glomerular filtration rate; SGLT2, sodium‐glucose cotransporter 2.

**FIGURE 1:**
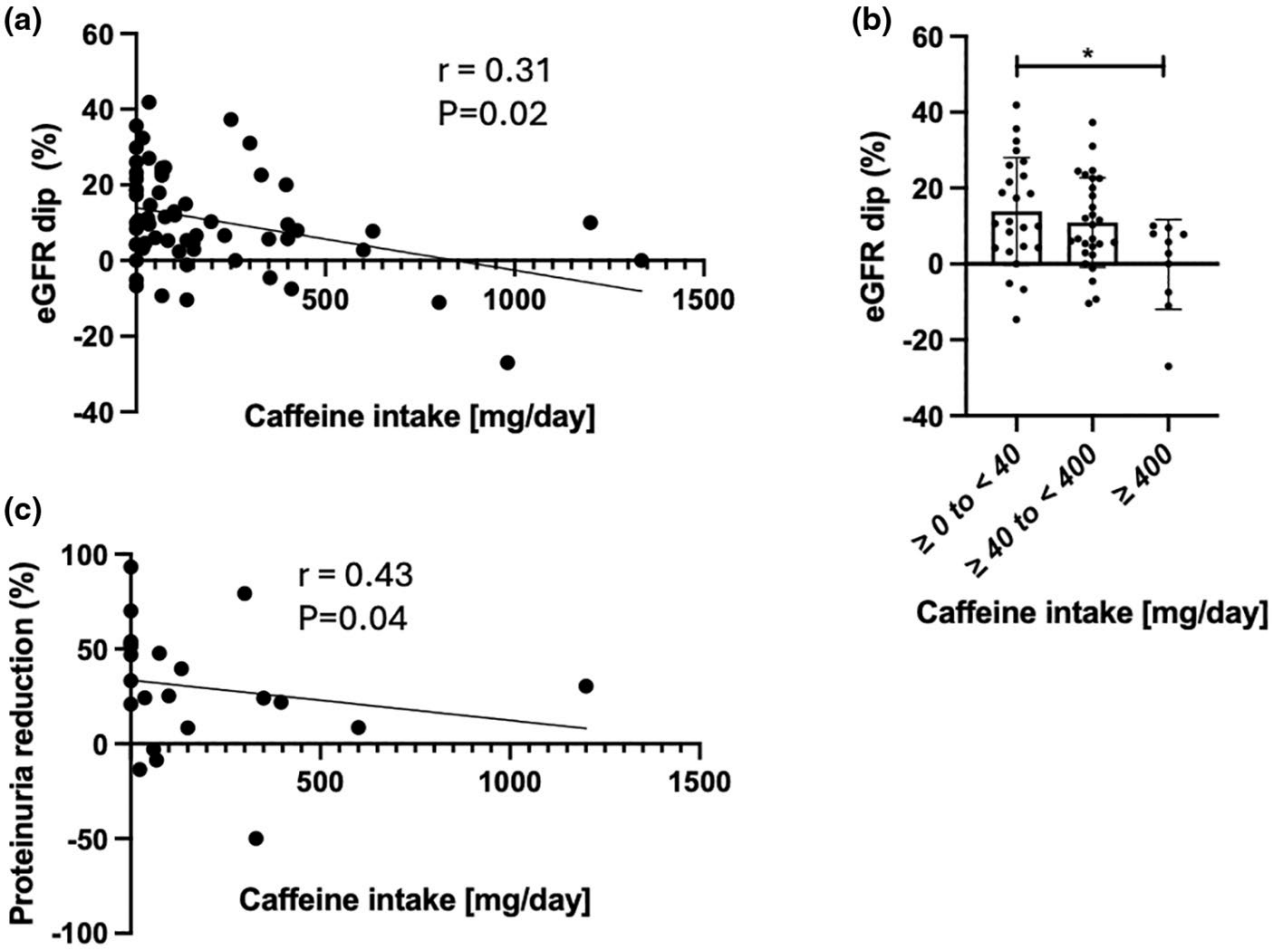
Correlation between caffeine intake and eGFR dip and proteinuria reduction. (a) Correlation between the daily caffeine intake (mg/day) and eGFR dip (%) at 1 month (*N* = 62). (b) eGFR dip (%) at 1 month in three groups of caffeine intake: No or small caffeine takers (≥0 to <40 mg/day, *N* = 24), moderate caffeine takers (≥40 to <400 mg/day, *N* = 28), and high caffeine takers (≥400 mg/day, *N* = 10). Data are expressed in mean ± SD, analyzed by Kruskal–Wallis test. (c) Correlation between the daily caffeine intake (mg/day) and proteinuria reduction (%) at 1 month (*N* = 22). *r* = correlation coefficient by Spearman correlation analysis.

As shown in Figure 1a, eGFR dip at 1 month after SGLT2i initiation was negatively correlated with caffeine intake (*N* = 62, *r* = −0.31, *p* = 0.02). When participants were divided into three groups: no or small caffeine takers (≥0 to <40 mg/day), moderate caffeine takers (≥40 to <400 mg/day), and high caffeine takers (≥400 mg/day), as defined in previous literature (Zhang et al., 2023), the high caffeine takers had significantly reduced eGFR dip (Figure 1b). For those in whom proteinuria data were available at 1 month, proteinuria reduction at 1 month was also negatively correlated with caffeine intake (Figure 1c, *N* = 22, *r* = −0.43, *p* = 0.04).

To identify variables independently associated with eGFR dip, regression analyses were performed. We assessed caffeine intake as well as clinical variables that are reportedly associated with larger eGFR dips, such as older age (Adamson et al., 2022), type 2 diabetes (Adamson et al., 2022), diuretic use (Kraus et al., 2021; Mayer et al., 2019), renin‐angiotensin‐aldosterone system inhibitor (RAASi) use (Mayer et al., 2019), higher systolic blood pressure (Kraus et al., 2021), and lower (Adamson et al., 2022) or higher (Shibata et al., 2023) eGFR. In our cohort, serum creatinine level and caffeine intake were negatively correlated with a larger eGFR dip. Furthermore, multivariable regression analysis revealed that these variables were independently associated with eGFR dip (Table 2).

**TABLE 2:** Univariable and multivariable regression analyses for the determinants of initial eGFR dip.

Variables	Univariable regression		Multivariable regression^a^	
	β	*p* value	*β*	*p* value
Age, years	−1.38805	0.6797		
Gender (male)	−0.07138	0.6008		
DM	3.90162	0.3300		
Serum Cre, mg/dL	−7.82119	0.0249*	−10.13117	0.002***
eGFR, mL/min/1.73 m^2^	0.12716	0.0567		
Caffeine intake, mg/day	−0.01649	0.004***	−0.01978	0.0004***
Diuretic use	−3.30604	0.3238		
RAASi use	4.48199	0.2234		
SBP, mmHg	−0.04726	0.6553		

Abbreviations: Cre, creatinine; DM, diabetes mellitus; RAASi, renin–angiotensin–aldosterone system inhibitors; SBP, systolic blood pressure.

^a^ Variables entered in the model: serum Cre and caffeine intake.

^*^ *p* < 0.05.

^***^ *p* < 0.005.

## 4 DISCUSSION

Our retrospective study shows that high caffeine intake may be associated with an attenuated initial eGFR dip following the initiation of an SGLT2 inhibitor. Proteinuria reduction at 1 month after SGLT2i was also negatively correlated with caffeine intake, which is in line with previous studies suggesting that eGFR dip is correlated with proteinuria reduction by SGLT2i (Shimizu et al., 2024). These findings are consistent with a decreased change in intraglomerular pressure by SGLT2i initiation in high caffeine takers.

Kidokoro et al. showed that an A1 adenosine receptor antagonist attenuated empagliflozin's effect of decreasing single‐nephron GFR in type 1 diabetic Akita mice, suggesting that adenosine signaling may be a key regulator of the glomerular hemodynamic change with SGLT2i (Kidokoro et al., 2019). Caffeine is a nonselective adenosine receptor antagonist, and it can block the signal transmission in the TGF mechanism, similar to an A1 adenosine receptor antagonist. Therefore, it is plausible that caffeine's ability to blunt the eGFR dip in SGLT2i recipients may be from preventing the constriction of the afferent arteriole via blocked TGF.

Another potential mechanism involves caffeine's inhibition of Na^+^/H^+^ exchanger isoform 3 (NHE3) (Fenton et al., 2015). The modulation of NHE3 activity by SGLT2i is thought to play a crucial role in proximal tubular sodium handling (Onishi et al., 2020). Consequently, under conditions of chronic NHE3 suppression by caffeine, sodium delivery to the macula densa may not increase after SGLT2i initiation, potentially nullifying its impact on eGFR dipping. However, a role of NHE3 in the diuretic response to caffeine in mice has been challenged (Fenton et al., 2015).

Some limitations in our study should be noted. Because of the retrospective nature of this study, it only determines association, not causation. Type 2 errors are possible given the small sample size of high caffeine takers. There is also a possibility of recall bias as a few of the patients were started on SGLT‐2i long before taking our survey, although there was no difference in time between the SGLT2i initiation and the survey among the three groups based on caffeine intake. Moreover, we could not confirm adherence to RAASi and SGLT2i throughout the study period.

Although it remains uncertain to what extent the renal hemodynamic effect of SGLT2i contributes to improved renal outcomes, caffeine intake, at least partly, may modulate the hemodynamic changes after SGLT2i initiation. Further study is needed to confirm an influence of high caffeine intake on the initial eGFR response and evaluate its long‐term effects on SGLT2 inhibitor's renoprotective properties.

## AUTHOR CONTRIBUTIONS

**Zach Kisley** and **Kana N. Miyata**: conceived and designed research. **Zach Kisley**, **Amanda J. Kalishman**, **Aliza A. Memon**, **Sandeep S. Dhindsa**, **John C. Edwards**, **Amy Mosman**, **Krista L. Lentine**, **Paul Kunnath**, **Emily Wood**, and **Kana N. Miyata** performed experiments. **Zach Kisley** and **Kana N. Miyata**: analyzed data. **Sandeep S. Dhindsa**, **John C. Edwards**, **Krista L. Lentine** and **Kana N. Miyata**: interpreted results of experiments. **Kana N. Miyata** prepared figures. **Zach Kisley** drafted manuscript. **Amanda J. Kalishman**, **Aliza A. Memon**, **Sandeep S. Dhindsa**, **John C. Edwards**, **Krista L. Lentine** and **Kana N. Miyata** edited and revised manuscript. All the authors approved final version of the manuscript.

## FUNDING INFORMATION

Kana N. Miyata was supported by the Bander Foundation grant 2022–2023. Krista L. Lentine was supported by the Mid‐America Transplant/Jane A. Beckman Endowed Chair in Transplantation.

## CONFLICT OF INTEREST STATEMENT

Outside of this work, Krista L. Lentine received consulting fees from CareDx and speaker honoraria from Sanofi, and Sandeep S. Dhindsa received honoraria from Bayer, Acerus, Tolmar, Verity and Marius pharmaceuticals, and research support from Clarus Therapeutics. The other authors have no relevant interests to disclose.

## ETHICS STATEMENT

This study was reviewed and approved by the Saint Louis University Institutional Review Board and was conducted in accordance with the Declaration of Helsinki.

## Data Availability

The data set is available from the corresponding author upon request.
